# Elevated Temperature and Exposure to Copper Leads to Changes in the Antioxidant Defense System of the Reef-Building Coral *Mussismilia harttii*

**DOI:** 10.3389/fphys.2021.804678

**Published:** 2021-12-23

**Authors:** Juliana da Silva Fonseca, Laura Fernandes de Barros Marangoni, Joseane Aparecida Marques, Adalto Bianchini

**Affiliations:** ^1^Programa de Pós-Graduação em Ciências Fisiológicas, Instituto de Ciências Biológicas, Universidade Federal do Rio Grande, Rio Grande, Brazil; ^2^Programa de Pós-Graduação em Oceanografia Biológica, Instituto de Oceanografia, Universidade Federal do Rio Grande, Rio Grande, Brazil; ^3^Instituto Coral Vivo, Santa Cruz Cabrália, Brazil; ^4^Smithsonian Tropical Research Institute, Ciudad de Panamá, Panama; ^5^Instituto de Ciências Biológicas, Universidade Federal do Rio Grande, Rio Grande, Brazil

**Keywords:** coral bleaching, coral reefs, global warming, metal pollution, antioxidant enzymes, oxidative state

## Abstract

The frequency and severity of coral bleaching events have increased in recent years. Global warming and contamination are primarily responsible for triggering these responses in corals. Thus, the objective of this study was to evaluate the isolated and combined effects of elevated temperature and exposure to copper (Cu) on responses of the antioxidant defense system of coral *Mussismilia harttii*. In a marine mesocosm, fragments of the coral were exposed to three temperatures (25.0, 26.6, and 27.3°C) and three concentrations of Cu (2.9, 5.4, and 8.6 μg/L) for up to 12 days. Levels of reduced glutathione (GSH) and the activity of enzymes, such as superoxide dismutase (SOD), catalase (CAT), glutathione S-transferase (GST), and glutamate cysteine ligase (GCL), were evaluated on the corals and symbionts. The short exposure to isolated and combined stressors caused a reduction in GSH levels and inhibition of the activity of antioxidant enzymes. After prolonged exposure, the combination of stressors continued to reduce GSH levels and SOD, CAT, and GCL activity in symbionts and GST activity in host corals. GCL activity was the parameter most affected by stressors, remaining inhibited after 12-days exposure. Interesting that long-term exposure to stressors stimulated antioxidant defense proteins in *M. harttii*, demonstrating a counteracting response that may beneficiate the oxidative state. These results, combined with other studies already published suggest that the antioxidant system should be further studied in order to understand the mechanisms of tolerance of South Atlantic reefs.

## Introduction

Ocean warming is the main factor causing bleaching in coral reefs worldwide (Hoegh-Guldberg, 1999; McWilliams et al., 2005; Yuyama et al., 2012). Bleaching can be defined as the loss of color in the coral tissue, which can result from the expulsion of photosynthetic dinoflagellate endosymbionts from its tissues or degradation of photosynthetic pigments (Glynn, 1993; Downs et al., 2005). Under high stress conditions, reactive oxygen species (ROS) produced by symbionts can be passed to host coral tissues (Lesser, 1997, 2006; Weis, 2008; Yakovleva et al., 2009). In an attempt to avoid oxidative damage, corals eliminate an important source of ROS (their symbionts) what ultimately leads to the breakdown of the symbiotic relationship between the coral host and their photosynthetic symbionts (Downs et al., 2005; Howells et al., 2016). Due to the importance of such symbiosis, the bleaching is directly related to episodes of mass mortality on coral reefs, which contributes to the degradation of this important ecosystem (Glynn, 1993, 1996; Hoegh-Guldberg, 1999; Suggett and Smith, 2019).

The symbiosis between corals and photosynthetic algae produces chronic hyperoxic conditions during the light hours (Dykens, 1984; Kuhl et al., 1995; Schwarz et al., 2013). To protect themselves against potential damage induced by ROS, corals, and symbionts contain a variety of molecules with antioxidant properties (Lesser, 2006). The antioxidant defense system (ADS) includes enzymatic and non-enzymatic molecules, which reduce the amount of ROS and maintain the balance of the cellular oxidative state (Monteiro et al., 2006; Suggett and Smith, 2019). Antioxidant enzymes, such as superoxide dismutase (SOD), catalase (CAT), glutathione S-transferase (GST), and glutamate cysteine ligase (GCL), are responsible for acting in the neutralization of ROS. In fact, increased activity of such enzymes in corals is related to higher concentrations of ROS (Lesser et al., 1990; Higuchi et al., 2015). Reduced glutathione (GSH) has non-enzymatic antioxidant activity acting as substrate for the GST and glutathione peroxidase (GPx; Hermes-Lima, 2004). Due to the important role of these molecules in redox metabolism, they can be used as interesting parameters to assess an early warning response to coral bleaching in the face of multiple stressors (Weis, 2008; Higuchi et al., 2015).

Metal exposure is typically known to induce oxidative stress in many organisms through its participation in biochemical reactions that produce ROS, such as the superoxide anion (O_2_^•−^), hydrogen peroxide (H_2_O_2_), and hydroxide radical (^•^OH; De Nadal et al., 2011; Schwarz et al., 2013). Copper (Cu) is a metal commonly found in the aquatic environment (Maria and Bebianno, 2011). This metal is considered an important micronutrient for the proper functioning of physiological systems, such as electron transport chain and antioxidant enzymes (Mercer and Llanos, 2003; Leary et al., 2009). However, elevated concentrations of Cu can be found in the aquatic environment due to run-off, mining, anti-fouling paints, and sewage (Jones, 1997). In fact, Cu contamination has been pointed out as a notable local threat to coral reefs (van Dam et al., 2011; Marques et al., 2019). When present in high concentrations, this metal can accumulate in organisms in concentrations above their capacity to excrete or detoxify (Rainbow, 2002). Toxic effects caused by Cu have been extensively reported in corals, including damage to the energy metabolism (Fonseca et al., 2019), reduction in the expression of antioxidant enzymes (Schwarz et al., 2013), DNA damage (Schwarz et al., 2013; Marangoni et al., 2017), reduced activity of enzymes related to the calcification process (Bielmyer et al., 2010; Marangoni et al., 2017), and bleaching (Jones, 2004; Gissi et al., 2019).

Thermal stress and exposure to heavy metals, such as Cu, can act in isolation and result in serious consequences. However, the impacts on the health of the organisms are more likely to be the result of a combination of stressors (Negri and Hoogenboom, 2011; Banc-Prandi and Fine, 2019). For example, the combination of these stressors presented a synergic effect in the coral *Mussismilia harttii* that led to reduced photosynthetic capacity (Fonseca et al., 2017), as well as decreased activity of enzymes involved in the energy metabolism (Fonseca et al., 2019). Cu is known to exhibit high affinity for thiol groups (−SH) present in antioxidants (Hultberg et al., 2001; Geracitano et al., 2002). Additionally, increased temperature can reduce the tolerance of organisms to exposure to metals (Portner, 2001, 2002). Therefore, it can be expected that the combination of thermal stress and Cu exposure could lead to increased deleterious effects in the oxidative metabolism of corals.

Most studies that evaluate the ADS of corals have been conducted with isolated stressors (Downs et al., 2000; Grant et al., 2003; Mitchelmore et al., 2007; Higuchi et al., 2009, 2012, 2015; Schwarz et al., 2013; Krueger et al., 2015). To date, only one study has evaluated the combined effect of increased temperature and exposure to copper on SOD activity in corals (Banc-Prandi and Fine, 2019). Considering the paucity of information on the combined effects of these stressors (temperature and Cu) in parameters associated with the ADS in corals, the present study aimed to contribute with a better understanding in this topic by evaluating in further details the ADS of an important scleractinian coral species of South Atlantic reefs, *M. harttii*, subjected to the combination of relevant stressors affecting coral reefs worldwide. For this purpose, we evaluated the levels of GSH and the activity of the antioxidant enzymes SOD, CAT, GST, and GCL in *M. harttii* exposed to different levels of thermal stress, Cu concentrations, and the combination of both. This species of coral is endemic and important in the construction of coral reefs in the South Atlantic. In addition, *M. harttii* has been subjected to Cu contamination associated with mining and sewage (Francini-Filho et al., 2019; Marques et al., 2019).

## Materials and Methods

### Collection of Corals

Polyps from three colonies of the coral *M. harttii* were collected during July 2012 on the conservation area of the Municipal Natural Park of Recife de Fora (16°24′31"S; 038°58′39"W; Porto Seguro, Bahia, northeastern Brazil) and transported to the mesocosm facility of the Coral Vivo Project (Arraial d’Ajuda, Porto Seguro). Polyps were individualized, glued on ceramic plates using cyanoacrylate, and acclimated to the experimental conditions for 20 days. Coral samples were collected under the permission of the Brazilian Environmental Agency (permit #85926584; IBAMA/SISBIO).

### Coral Exposure in the Marine Mesocosm

Corals were kept in twenty seven 10 L tanks distributed according to three warming treatments [mean local temperature, and 1.5 and 2.5°C above the mean; that is, 25.0, 26.6, and 27.9°C, considering warming levels predicted to future climate scenarios issued by IPCC (2014)] and three Cu concentrations (0, 3, and 5 μg/L above the mean natural concentration, that is, 2.9, 5.4, and 8.6 μg/L). The concentrations of Cu used in the experiment are below or close the limits allowed by the international and Brazilian legislations for the protection of aquatic environments (CONAMA, 2005; EPA, 2005). All treatments were tested in triplicated tanks, where two *M. harttii* polyps, randomly distributed, were kept in each tank. After 4 and 12 days of exposure, polyps were sampled (between 5–6 pm/17–18 h) and immediately flash frozen for posterior laboratory analysis (see below).

In the mesocosm system, seawater is pumped through a continuous flow from the adjacent coral reef to four 5,000 L underground reservoirs to receive the desired temperature treatments before reaching the twenty seven 10 L tanks. Due to its direct connection to the reef environment, this system kept seawater conditions (e. g. natural daily variation of temperature, turbidity, salinity, pH, zooplankton availability) very similar to those in the natural environment. Temperature was monitored and controlled by an Arduino-based system (Reef Angel, Fremont) while two 15-kW submersible heaters maintained the temperature required for each treatment. Concomitantly, seawater captured from the reef environment was held in four pairs of 1,000- L underground sumps to receive stock solutions of Cu. Cu stock solutions were daily prepared (24 h before use) from a standard solution of CuCl_2_ (1 g/L Cu). The seawater that received the Cu treatments was mixed with seawater coming from the primary treatment sumps using peristaltic pumps (flow rate of 0.17 L/min) before reaching the 10-L tanks. Seawater from the secondary system (Cu contamination) accounted for 10% of the total seawater flow reaching the 10-L aquariums, while heated water flow accounted for 90%. Before returning to the ocean, water was filtered and sterilized in an additional underground sump where ultraviolet filters and activated carbon cartridges were installed. Details on the functioning of this mesocosm system can be found in Duarte et al. (2015).

### Seawater Collection and Analysis

Every 3 days seawater was collected from the test aquariums for monitoring concentrations of Cu and dissolved organic carbon (DOC) throughout the experiment. The concentrations of dissolved Cu were evaluated in filtered (0.45-mm mesh filter) water samples acidified with nitric acid (HNO_3_, 1% final concentration; SupraPur, Merck^®^, Germany). For this purpose, water samples were desalted using the method proposed by Nadella et al. (2009) and the concentrations of Cu were determined through Atomic Absorption Spectrophotometry with Graphite Furnace (PerkinElmer^®^, Waltham, United States). DOC concentration was assessed by Total Organic Carbon analyzer (Shimadzu^®^, Japan). Pluviometry was obtained from the local weather station (Veracel Celulose, Brazil). Daily, random tanks from each treatment combination were assessed for temperature, pH (HI 9124, Hanna Instruments^®^, United States), and salinity (optical refractometer ITREF 10, Instrutemp^®^, São Paulo, Brazil). Temperature measurements were performed using loggers installed inside the tanks and at the reef environment to continuously monitor the seawater temperature every 30 min.

### Sample Preparation

The antioxidant defense parameters were quantified in both coral host and endosymbiont microalgae fractions. Samples were prepared as described by Fonseca et al. (2021). Coral fragments were cut, homogenized on ice with a buffer for each analysis (1:1 w/v) using a sonicator (20 kHz, Sonaer Ultrasonics^®^, New York, United States). After homogenization, samples were centrifuged (2,530 *g*, 5 min, 4°C) to separate coral (supernatant) and symbiont (pellet) homogenates. Afterward, 200 μl of buffer was added to the pellet, which was sonicated (30 kHz) and used for analysis of the symbiotic microalgae. All results were normalized considering the protein concentration of the homogenates, which was quantified using a commercial kit based on the Bradford method.

### Determination of Parameters of the Antioxidant Defense System

To determine the activity of the antioxidant enzymes CAT, SOD, and GST, the samples were homogenized in a buffer containing 20 mm Tris Base, 1 mm EDTA, 1 mm dithiothreitol, 500 mm sucrose, 150 mm KCl, and 0.1 mm PMSF. pH was adjusted to 7.6. For determination of the GCL activity and quantification of GSH content, the samples were homogenized in Tris-EDTA buffer containing 100 mm Tris-HCl, 2 mm EDTA, and 5 mm MgCl_2_·6H_2_O. pH was adjusted to 7.75. All enzymatic assays were carried out at 25°C.

CAT activity was determined as described by Beutler (1975). The assay consists of the decomposition of H_2_O_2_ by CAT present in the sample in water (H_2_O) and oxygen (O_2_). The decomposition of H_2_O_2_ by CAT is monitored by the decrease of absorbance in 240 nm. The reaction was evaluated in quartz microplates containing the homogenate and reaction buffer (1 M tris base, 5 mm EDTA, and H_2_O_2_ (30%), pH 8.0). The results are expressed in units of CAT, which is defined as the amount of enzyme required to hydrolyze 1 μmol of H_2_O_2_ per min and per mg of protein.

SOD activity was assessed according to McCord and Fridovich (1969). This analysis reflects changes in superoxide anion (O_2_^•−^) concentrations through the oxidation of cytochrome C by xanthine oxidase. The SOD assay was conducted using a buffer solution containing 50 mm KH_2_PO_4_ (pH 7.8). Absorbance readings were performed at 550 nm. SOD activity was expressed in enzyme units, where one unit is the amount of enzyme needed to inhibit 50% of cytochrome c reduction/min/mg protein.

Glutathione-S-transferase (GST) assay was performed as described by Habig and Jakoby (1981) using a phosphate buffer solution (100 mm, pH 7.0), following the conjugation of 1 mm GSH (Sigma-Aldrich, United States) with 0.77 mm 1-chloro-2,4- dinitrobenzene (CDNB; Sigma-Aldrich, United States). GST activity was measured as the increment in absorbance at 340 nm and was expressed in μmol CDNB/min/mg protein.

GCL activity and GSH concentration were analyzed according to White et al. (2003). The reaction is based on the measure of fluorescence emitted by the conjugation reaction of glutamylcysteine and GSH through fluorochrome NDA (naphthalene-2,3-dicarboxyaldehyde). The fluorescence measurement is evaluated by the wavelength of 472 nm (excitation) and 528 nm (emission). The activity of the GCL is expressed in ηmol of GSH per hour and per mg of protein and the GSH concentration in mg of GSH per mg of protein.

### Statistical Analysis

The effects of elevated temperature and exposure to Cu on the activity of CAT, SOD, GST, GCL, and GSH content, on corals and symbionts, were evaluated using 2-way factorial ANOVA followed by the Fisher test for multiple comparisons. At each experimental time (4 and 12 days) we performed one ANOVA [fixed factors “temperature” (three levels, 25.0, 26.6 and 27.3°C), and “Cu” (three levels, 2.9, 5.4 and 8.6 μg/L)]. Data normality and homogeneity of variances were previously verified using the Shapiro-Wilk and Cochran C tests, respectively, and log-transformed when necessary. The confidence level adopted was 95% (*α* = 0.05).

## Results

### Seawater Physicochemical Parameters

The measured concentrations of dissolved Cu were 2.9 ± 0.7, 5.4 ± 0.9, 8.6 ± 0.3 μg/L, corresponding to the treatments of 0 (control), 3 and 5 μg/L above the mean natural concentration of 2.9 μg/L, respectively. Temperature levels were 25.0 ± 0.1 for control, 26.6 ± 0.1 and 27.3 ± 0.1°C, corresponding to the treatments of 1.5 and 2.5°C above ambient seawater temperature. The other physicochemical parameters analyzed in seawater are shown in Supplementary Table S1.

### Parameters of the Antioxidant Defense System

**Figure 1:**
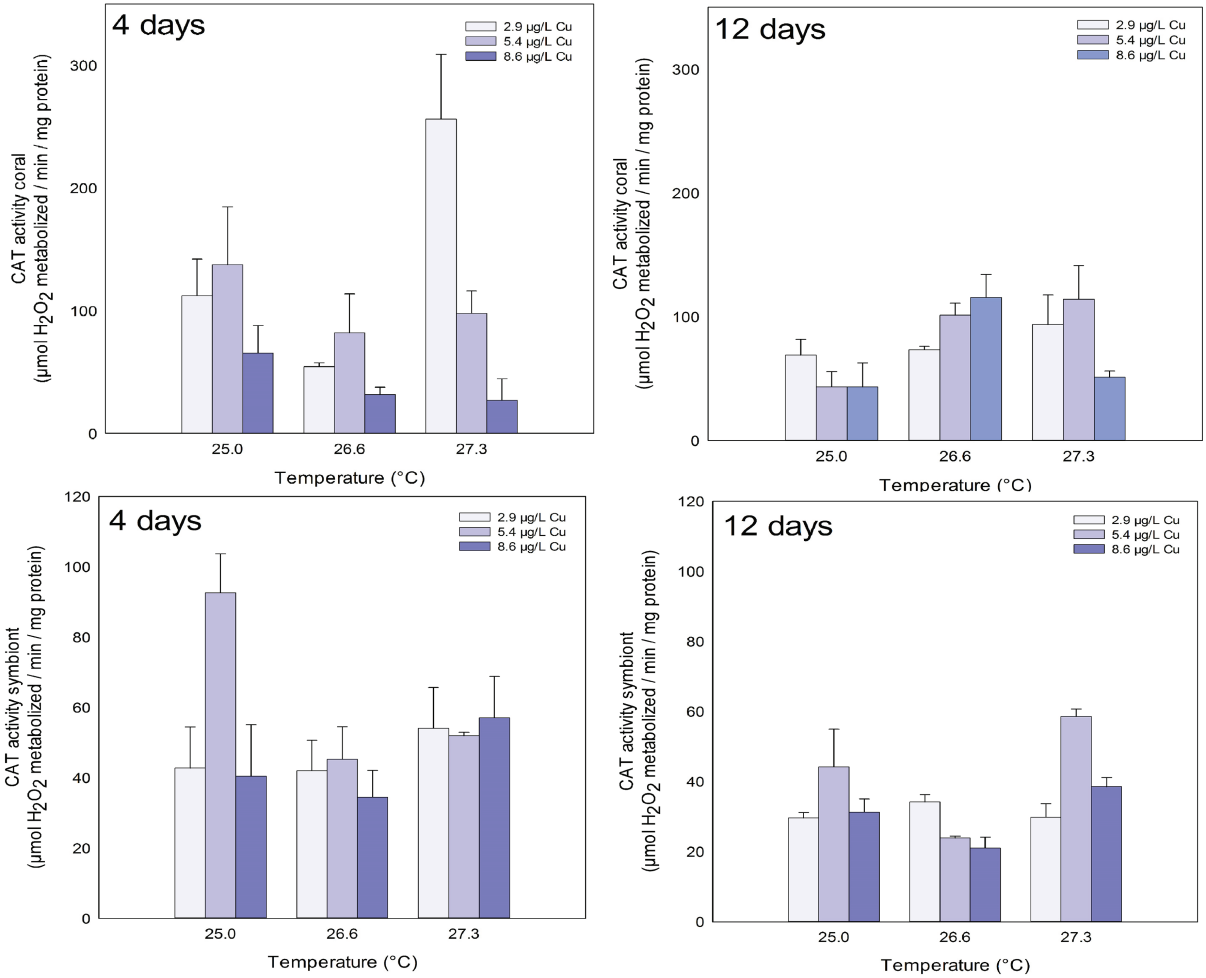
CAT activity in the coral and for the symbiotic algae of coral *Mussismilia harttii* exposed to three different temperatures and three different copper (Cu) concentrations for 4 and 12 days.

CAT activity in the coral host fraction was inhibited after 4 days of exposure in the highest concentration of Cu tested (8.6 μg/L). In turn, after 12 days of exposure, an increased activity of CAT was observed in the temperature treatments of 26.6 and 27.3°C compared to the ones maintained at 25°C (Figure 1; Table 1).

**Table 1:** Results for the 2-way factorial analysis of variance (ANOVA) conducted for *Mussismilia harttii* exposed to elevated temperature and Cu contamination for 4 and 12 days.

Variable	Treatment	4-days				12-days			
		*df*	*F*	*p*	Fisher	*df*	*F*	*p*	Fisher
CAT coral	Cu	2	11.94	0.00	^*^ **8.6 < 2.9, 5.4**	2	0.56	0.58	
	Temperature	2	2.89	0.08		2	4.65	0.02	**27.3, 26.6 > 25.0**
	Cu × Temperature	4	2.14	0.11		4	2.07	0.13	
CAT symbiont	Cu	2	3.11	0.06		2	4.23	0.03	^*^ **5.4 > 2.9, 8.6**
	Temperature	2	2.49	0.11		2	9.70	0.00	**26.6 < 25.0, 27.3**
	Cu × Temperature	4	2.68	0.06		4	4.51	0.01	**26.6 (8.6 < 2.9)**
									**27.3 (5.4 > 2.9, 8.6)**
SOD coral	Cu	2	0.80	0.46		2	0.60	0.55	
	Temperature	2	3.02	0.07		2	5.62	0.01	**26.6 > 25.0**
	Cu × Temperature	4	5.23	0.00	**27.3 (5.4 > 2.9, 8.6)**	4	0.93	0.46	
SOD symbiont	Cu	2	11.30	0.00	**5.4 > 2.9, 8.6**	2	6.90	0.00	^*^ **8.6 > 2.9**
	Temperature	2	17.39	0.00	**26.6 < 25.0 < 27.6**	2	5.34	0.01	**27.3, 26.6 > 25.0**
	Cu × Temperature	4	1.65	0.20		4	13.00	0.00	**25.0 (8.6 > 2.9, 5.4)**
									**26.6 (8.6 > 2.9, 5.4)**
									**27.3 (8.6 < 2.9, 5.4)**
GST coral	Cu	2	2.33	0.12		2	0.53	0.59	
	Temperature	2	2.03	0.16		2	50.22	0.00	**26.6 > 25.0, 27.3**
	Cu × Temperature	4	1.10	0.38		4	3.10	0.04	**26.6 (8.6 > 2.9)**
									**27.6 (8.6 < 2.9)**
GST symbiont	Cu	2	6.21	0.00	**5.4 > 8.6**	2	1.36	0.28	
	Temperature	2	7.87	0.00	**26.6 < 25.0, 27.3**	2	2.75	0.09	
	Cu × Temperature	4	1.05	0.40		4	0.71	0.59	
GCL coral	Cu	2	11.47	0.00	**5.4 < 2.9, 8.6**	2	14.09	0.00	^*^ **8.6 < 2.9, 5.4**
	Temperature	2	0.68	0.52		2	23.55	0.00	**26.6 < 25.0, 27.3**
	Cu × Temperature	4	4.29	0.02	**26.6 (5.4 < 2.9)**	4	2.53	0.09	
					**27.3 (5.4, 8.6 < 2.9)**				
GCL symbiont	Cu	2	3.10	0.07		2	2.38	0.13	
	Temperature	2	4.21	0.03	^*^ **27.6, 26.6 < 25.0**	2	3.39	0.06	
	Cu × Temperature	4	1.93	0.16		4	3.40	0.04	^*^ **25.0 (8.6 < 5.4)**
									**27.0 (5.4 < 2.9)**
GSH coral	Cu	2	1.61	0.22		2	4.09	0.03	**5.4 > 2.9**
	Temperature	2	1.67	0.21		2	8.38	0.00	**27.3 > 26.6, 25.0**
	Cu × Temperature	4	4.25	0.01	**25.0 (5.4 < 2.9, 8.6)**	4	2.40	0.09	
					**26.6 (8.6 < 5.4)**				
GSH symbiont	Cu	2	14.58	0.00	**5.4 < 2.9, 8.6**	2	1.89	0.31	
	Temperature	2	6.29	0.01	**26.6 > 25.0, 27.3**	2	1.26	0.18	
	Cu × Temperature	4	7.28	0.00	**26.0 (5.4 < 2.9)**	4	4.88	0.01	**25.0 (8.6 < 2.9, 5.4)**
									**26.6 (5.4 > 2.9)**

Significant values (*p* < 0.05) are in bold. The results marked in green represent an increase in the ADS, while the results marked in red represent the inhibitory effects on the ADS of M. harttii.

* indicates the data that has been log-transformed.

No effects were observed in the CAT activity of symbionts after 4 days of exposure. However, effects of increased temperature, Cu, and the combination of both were observed after 12 days. Exposure to 5.4 μg/L Cu increased CAT activity on symbionts compared to 2.9 and 8.6 μg/L Cu. Exposure to 26.6°C reduced CAT activity compared to 25.0 and 27.3°C. In addition, the results of the combination of stressors showed an inhibition of CAT activity under 26.6°C and 8.6 μg/L compared to 2.9 μg/L at the same temperature. Exposure to 27.3°C and 5.4 μg/L increased CAT activity in symbionts compared to those maintained at 2.9 and 8.6 μg/L at the same temperature (Figure 1; Table 1).

**Figure 2:**
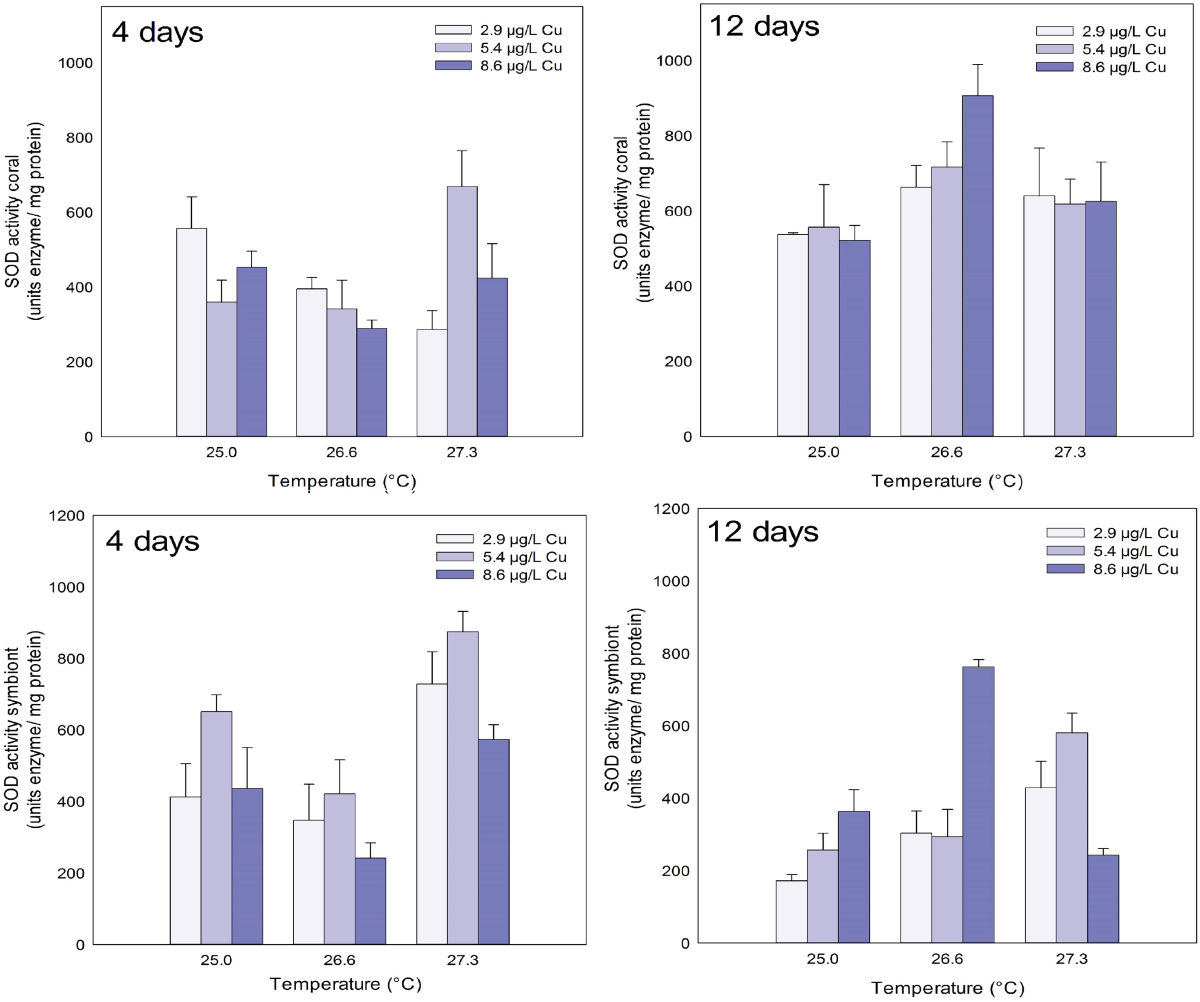
SOD activity in the coral and for the symbiotic algae of coral *M. harttii* exposed to three different temperatures and three different copper (Cu) concentrations for 4 and 12 days.

SOD activity in the coral host increased after 4 days of exposure to the combination of stressors. An increased activity of the enzyme was observed under 27.3°C and 5.4ug/L Cu compared to 2.9 and 8.6ug/L Cu at the same temperature. After 12 days, exposure to 26.6°C led to an increased activity of SOD compared to 25.0°C (Figure 2; Table 1).

Effects of temperature and Cu applied in isolation were observed on SOD activity in symbionts after 4 days of exposure. Exposure to the concentration of 5.4 μg/L Cu increased SOD activity compared to 2.9 and 8.6 μg/L Cu. Exposure to 26.6°C inhibited SOD activity in symbionts compared to 25.0°C. In turn, exposure to 27.3°C increased SOD activity in symbionts compared to 25.0 and 26.6°C. After 12 days, the effect of exposure to isolated and combined stressors was observed on SOD activity in symbionts. A significant increase at 8.6 μg/L C compared to 2.9 μg/L Cu was observed. An increase was also observed under 26.6°C and 27.3°C compared to the control temperature (25.0°C). The combination of stressors led to an increase in SOD activity under 25.0 and 26.6°C combined with 8.6 μg/L Cu compared to 2.9 and 5.4 μg/L Cu at the same temperatures. Exposure to 27.3°C and 8.6 μg/L reduced SOD activity in symbionts compared to those maintained at 2.9 and 5.4 μg/L at the same temperature (Figure 2; Table 1).

**Figure 3:**
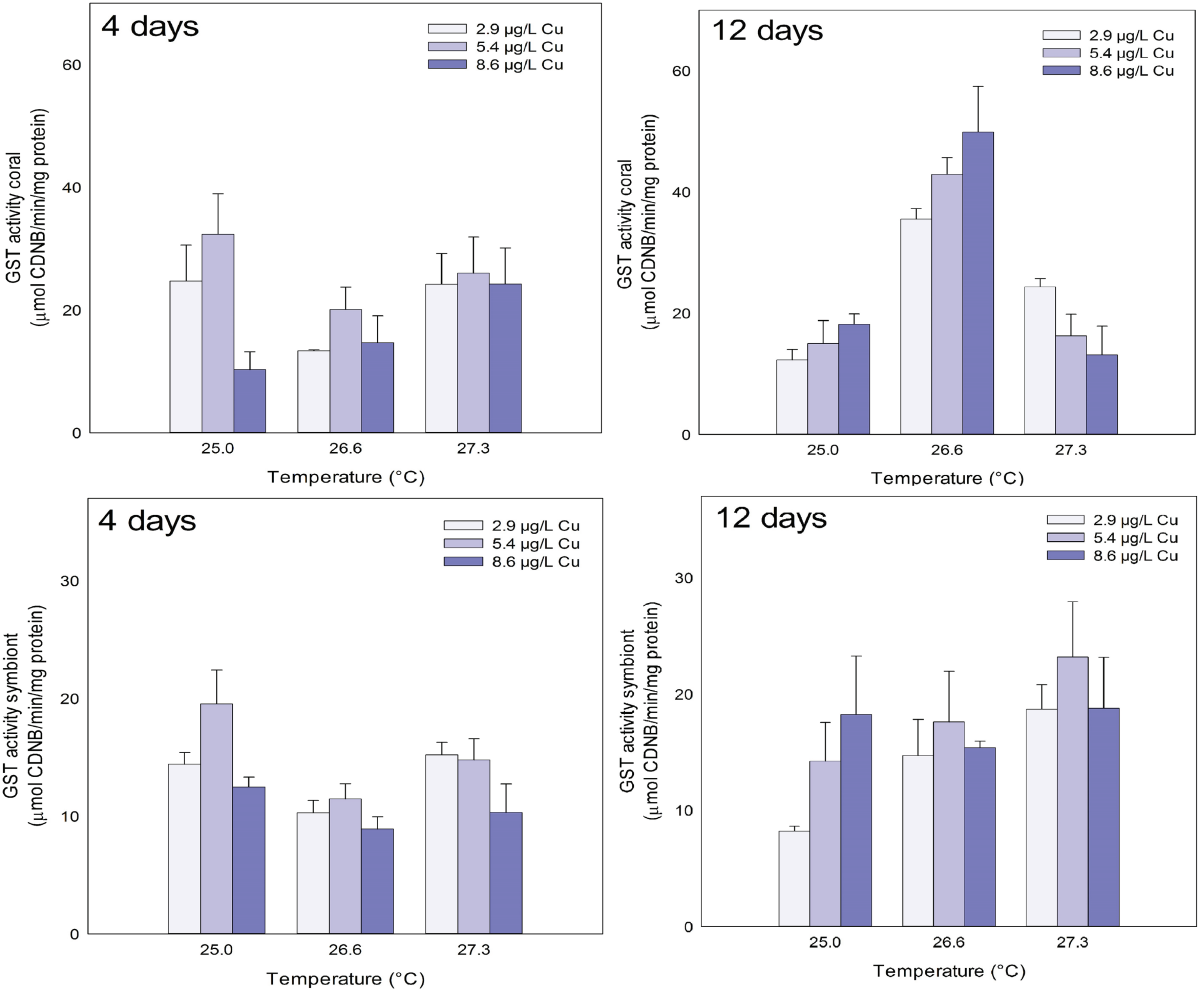
GST activity in the coral and for the symbiotic algae of coral *M. harttii* exposed to three different temperatures and three different copper (Cu) concentrations for 4 and 12 days.

No effects were observed in GST activity for any of the treatments tested on corals after 4 days. However, after 12 days, effects of temperature and the combination of stressors were observed on GST activity in the coral host. Exposure to 26.6°C increased GST activity compared to 25.0 and 27.3°C. In the combined exposure, an increase in GST activity was observed under 26.6°C and 8.6 μg/L compared to 2.9 μg/L Cu at the same temperature. However, corals maintained at 27.3°C and 8.6 μg/L Cu showed an inhibition of GST activity compared to those maintained at the same temperature at 2.9 μg/L Cu (Figure 3; Table 1).

Effects of Cu and temperature were observed on GST activity in symbionts after 4 days of exposure. Exposure to 5.4 μg/L Cu increased GST activity compared to 8.6 μg/L Cu. The opposite was observed for temperature. Exposure to 26.6°C inhibited GST activity compared to 25.0 and 27.3°C. No effects were observed in the symbiont GST activity after 12 days of exposure (Figure 3; Table 1).

**Figure 4:**
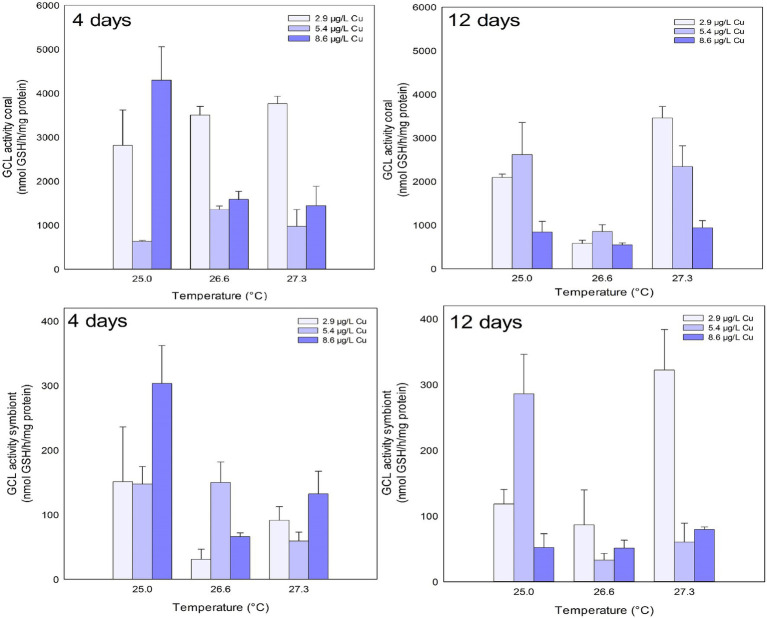
GCL activity in the coral and for the symbiotic algae of coral *M. harttii* exposed to three different temperatures and three different copper (Cu) concentrations for 4 and 12 days.

GCL activity in the coral host was inhibited after 4 days of exposure to Cu and the combination of stressors. Exposure to 5.4 μg/L Cu inhibited GCL activity compared to 2.9 and 8.6 μg/L Cu. An inhibition was also observed under 26.6°C and 5.4 μg/L Cu compared to 2.9 μg/L Cu at the same temperature. Exposure to 27.3°C and 5.4 μg/L and 8.6 μg/L Cu also reduced GCL activity in corals compared to those maintained at 2.9 μg/L Cu at the same temperature. The isolated effects of temperature and Cu exposure were observed on GCL activity in the coral host after 12 days. Exposure to 8.6 μg/L Cu inhibited GCL activity compared to 2.9 and 5.4 μg/L Cu. Similarly, exposure to 26.6°C inhibited GCL activity in corals compared to 25.0 and 27.3°C (Figure 4; Table 1).

The isolated effects of temperature were observed for GCL activity in symbionts after 4 days. Exposure to 26.6 and 27.3°C inhibited GCL activity in symbionts compared to 25.0°C. After 12 days, combined exposure to stressors inhibited GCL activity in symbionts. The results indicate a significant reduction after exposure to high concentration of Cu (8.6 μg/L) compared to 5.4 μg/L Cu. A reduction was also observed under 27.3°C and 5.4 μg/L Cu compared to 2.9 μg/L Cu at the same temperature (Figure 4; Table 1).

**Figure 5:**
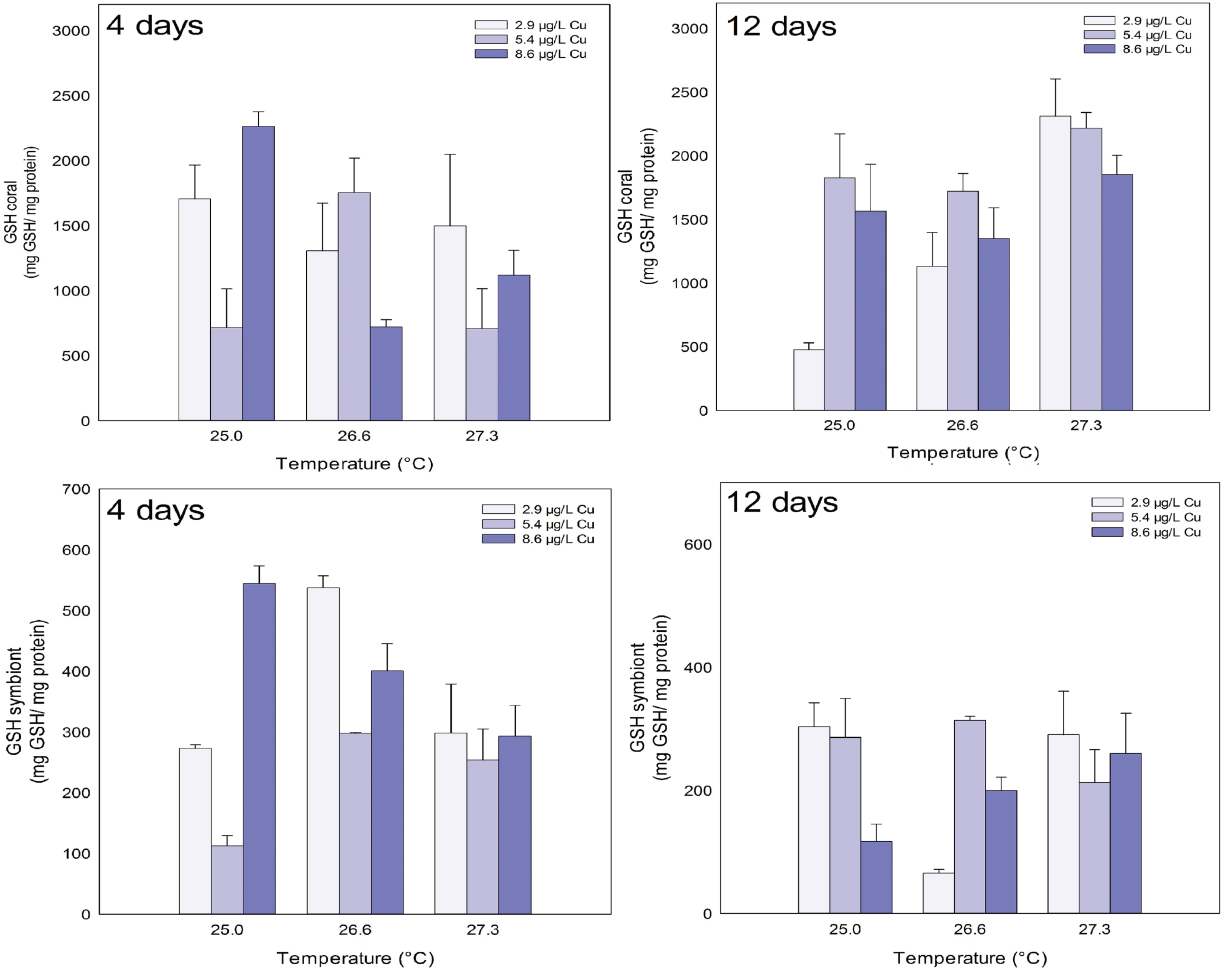
GSH levels in the coral and for the symbiotic algae of coral *M. harttii* exposed to three different temperatures and three different copper (Cu) concentrations for 4 and 12 days.

The levels of GSH in the coral host were reduced after 4 days of exposure to the combination of stressors. A significant reduction was observed after exposure to Cu at 5.4 μg/L compared to 2.9 and 8.6 μg/L. A reduction was also observed under 26.6°C and 8.6 μg/L Cu compared to 5.4 μg/L Cu at the same temperature. After 12 days, the isolated effects of temperature and Cu increased the levels of GSH in coral host. Exposure to 5.4 μg/L Cu increased the levels of GSH in the corals compared to those exposed to 2.9 μg/L Cu. Exposure to 27.3°C increased the levels of GSH in corals compared to those exposed 25.0 and 26.6°C (Figure 5; Table 1).

Effects of temperature, Cu, and the combination of both were observed on the GSH levels of the symbionts after 4 days of exposure. Exposure to 5.4 μg/L Cu reduced GSH levels in symbionts compared to 2.9 and 8.6 μg/L Cu treatments. In turn, exposure to 26.6°C increased GSH levels in symbionts compared to 25.0 and 27.3°C. The combined treatment of 26.6°C and 5.4 μg/L Cu reduced the levels of GSH in the symbionts compared to 2.9 μg/L Cu at the same temperature. After 12 days, the combination of stressors altered the levels of GSH in the symbionts. Specifically, a reduction after exposure to Cu alone at 8.6 μg/L was observed compared to the treatments of 2.9 and 5.4 μg/L Cu. In turn, an increase in GSH levels was observed under 26.6°C and 5.4 μg/L Cu compared to 2.9 μg/L Cu at the same temperature (Figure 5; Table 1).

## Discussion

Oxidative stress is defined as an imbalance in the pro-oxidant/antioxidant ratio, which favors increased pro-oxidants and results in oxidative damage (Downs et al., 2002; Halliwell and Gutteridge, 2007). Previous studies have shown that the elevated temperature and exposure to Cu are typically known to induce oxidative damage in *M. harttii* (Fonseca et al., 2017; Marangoni et al., 2017, 2019a,b; Fonseca et al., 2021), as well as in other coral species (Lesser, 1997; Schwarz et al., 2013; Dias et al., 2019). Given the clear connection between oxidative stress and coral bleaching (for a review see Suggett and Smith, 2019), it is paramount to evaluate changes in the activity of specific antioxidant molecules that are associated with this condition. In this context, the present study was carried out evaluating a higher number of parameters related to ADS (enzymatic and non-enzymatic) than previous studies conducted with reef-building corals. A larger picture of the oxidative profile of a coral species was obtained in the face of the combined exposure of global and local stressors.

Exposure to increased temperature and Cu generally caused an inhibition in the activity of antioxidant enzymes after short exposure (4 days). Interestingly, after prolonged exposure (12 days) many of these parameters recovered and an increase in the antioxidant defenses of the coral host and its symbionts was observed. Results suggest that prolonged exposure to stressors can increase ROS generation in *M. harttii*. However, the coral holobiont (coral host + microalgae symbionts) showed a counteracting response under such condition by increasing antioxidant defenses to prevent oxidative damage. In fact, Fonseca et al. (2017) showed a reduction in lipid damage (as lipid peroxidation) in *M. harttii* exposed for up to 12 days under the same experimental conditions. Later, an increase in total antioxidant capacity (TAC) in algae symbionts of *M. harttii* was observed after exposure to increasing temperature and Cu (Fonseca et al., 2021). Interestingly, South Atlantic corals have been suggested to be more tolerant to stressors compared to coral reefs in other parts of the world (Mies et al., 2020). The up-regulation shown by *M. harttii* ADS and results found for the same specie under a heat wave event (Marangoni et al., 2019a,b) suggest that the antioxidant response is an important mechanism potentially connected to the resilience of these corals and indicate that this process should be further studied in order to understand the traits that explain the tolerance of the South Atlantic corals.

SOD and CAT are enzymes involved with the neutralization of ROS and their performance have been related to coral bleaching (Higuchi et al., 2015). The high activity of SOD and CAT after prolonged exposure of the coral host to increasing temperature suggests an increased need for neutralization of superoxide anion (O_2_^•−^), a radical with high potential of inducing oxidative damage. The dismutation of O_2_^•−^ performed by SOD represents an important source of H_2_O_2_, a reactive species mainly reduced by CAT (Regoli and Giuliani, 2014). Therefore, a higher generation of O_2_^•−^ would be expected after prolonged exposure of *M. harttii* to increasing temperature.

The symbiotic algae demonstrated an increase in SOD activity and a reduction in CAT after prolonged exposure to increasing temperature alone and combined with Cu. This can cause an accumulation of H_2_O_2_ in the symbiont cell. The excess of H_2_O_2_ can facilitate its reaction with transition metals, such as Fe and Cu, through the Fenton reactions facilitating the formation of hydroxyl radical (^•^OH; H_2_O_2_ + Fe^2+^/Cu^+^ → Fe^3+^/Cu^2+^ + ^•^OH + OH^−^, see Hermes-Lima, 2004). In addition, high amounts of H_2_O_2_ can induce apoptosis and the formation of nitrite peroxide (ONOO^−^; Perez and Weis, 2006; Weis, 2008; Hawkins et al., 2013; Hawkins and Davy, 2013), a highly reactive nitrogen species (RNS) that has been linked to the onset of coral bleaching (Marangoni et al., 2019b). RNS, such as ONOO^−^, are generally responsible for causing damage to proteins and DNA, in addition to inhibiting key enzymes in energy metabolism (Wink and Mitchell, 1998; Brown and Borutaite, 2002). In accordance, the same stressors tested here caused drastic reductions in enzymes of the energy metabolism of *M. harttii* after prolonged exposure (Fonseca et al., 2019). Cu acts as a cofactor of SOD (Hermes-Lima, 2004), therefore, the higher concentrations of such metal may have acted as an important factor inducing SOD activity in symbionts. In accordance, the increase in SOD activity has already been reported in corals exposed to Cu (Banc-Prandi and Fine, 2019).

GST is an enzyme of the phase II of the detoxification processes responsible for conjugating GSH with many organic and inorganic compounds, including aldehydes produced during lipid peroxidation (LPO; Higgins and Hayes, 2011). In fact, it has been shown that exposure to increased temperature alone induces LPO in host corals after prolonged exposure (Fonseca et al., 2021). This result was paralleled to increased GST activity and increased GSH levels in corals. Interestingly, increased temperature reduced the levels of LPO in the symbionts (Fonseca et al., 2021) and no changes were observed in the present study in GST activity and GSH levels. These results suggest a significant role of the glutathione system in the detoxification of LPO metabolites in corals and symbionts.

Considering the glutathione metabolism, GCL is the rate-limiting enzyme in GSH synthesis (White et al., 2003). The activity of this enzyme was inhibited in corals and symbionts throughout the experiment, which resulted in the reduction of GSH levels. Those parameters were the most affected by exposure to stressors, and similar results have already been observed in other studies (Downs et al., 2000; Pourahmad and O’Brien, 2000; Nagalakshmi and Prasad, 2001; Klein et al., 2017a; Maher, 2018). GCL is an ATP-dependent enzyme (Chen et al., 2005), and Cu and increased temperature have been previously shown to inhibit enzymes of energy metabolism in *M. harttii* (Fonseca et al., 2019). GSH plays an important role in numerous intracellular processes and provides protection for cells from damage caused by ROS and other stressors (Halliwell and Gutteridge, 2007). It contains a thiol group (−SH) with important reducing capacity, binding to electrophilic compounds by assisting its elimination or biotransformation (Townsend et al., 2003). Therefore, the reduction of GSH levels can compromise detoxification reactions of organic compounds, as well as the oxidative state of corals. Also, it is worth noting that exposure to isolated and combined stressors increased GSH levels in some treatments. Interestingly, the increase in GSH levels was parallel to the inhibition of GCL activity in host corals and their symbionts. In fact, it has already been observed that GCL activity can be inhibited by high levels of GSH (Maher, 2005; Klein et al., 2017b).

Reef-building corals need to have fine control in H_2_O_2_ neutralization to prevent bleaching (Lesser, 1997; Smith et al., 2005; Weis, 2008). The results of the present study showed a possible relationship between the activity of SOD and GST and the levels of GSH in corals and symbionts. Prolonged exposure to elevated temperature increased the activity of SOD in corals concomitantly with the increase in GST activity and levels of GSH. This relationship may be involved with the conjugation of GSH with LPO products produced by accumulation of H_2_O_2_, which are catalyzed by GST. In fact, elevated temperature alone induced LPO in *M. harttii* (Fonseca et al., 2021). In addition, H_2_O_2_ can be decomposed by GPx oxidizing GSH in this process (Hermes-Lima, 2004), which could explain the increase in the production of this tripeptide. However, the symbiotic algae showed that the exposure to Cu alone induced SOD and GST activity after 4 days of exposure and this cause a depletion of GSH levels. In primary producers, the excess H_2_O_2_ generated in the presence of Cu is scavenged by peroxisomes. The detoxification pathway continues through the oxidation of GSH *via* the ascorbate-dehydroascorbate system (Nagalakshmi and Prasad, 2001). Thus, exposure to Cu can facilitate the accumulation of H_2_O_2_ in symbiotic algae and consequently trigger bleaching through this mechanism. In addition, GSH is known to be involved in metal scavenging (Hermes-Lima, 2004), so this can happen because GSH is facilitating the elimination of Cu in symbionts.

Symbiotic algae can be a major input of ROS to the coral host under stressful conditions, especially when it involves thermal stress and high light conditions (Suggett and Smith, 2019). Due to their photosynthetic nature, these organisms produce high amounts of ROS and therefore an efficient defense system to prevent oxidative damage can be expected. Indeed, exposure to stressors caused an increase in the parameters of the defense system of symbiotic algae. Increases in the TAC of low molecular weight scavengers have been observed in symbionts of *M. harttii* exposed to a combination of temperature and Cu (Fonseca et al., 2021). The up-regulation of antioxidant enzymes is an important component of the cellular response to stress of symbionts caused by exposure to stressors. This can provide the symbiotic algae a faster reduction of oxidative damage, prevent the process of apoptosis and consequently maintain the normal functioning of the photosynthetic process (Ralph et al., 2001). These results are interesting, as they reinforce that bleaching in *M. harttii* can be a response initiated by the symbiotic algae. Therefore, a response from the symbiotic algae defense system would be expected before any visual manifestation of bleaching. These results highlight the use of these parameters as potential tools of early warning response to corals bleaching.

Antioxidant enzyme analyzes are important and should be considered in future studies that evaluate the physiological responses of *M. harttii* coral under chronic exposure conditions. The evaluation of ADS parameters, preferentially combined with analysis of ROS and RNS, can improve our understanding about mechanisms of resilience in corals exposed to multiple stressors.

## Data Availability Statement

The original contributions presented in the study are included in the article/Supplementary Material, further inquiries can be directed to the corresponding author.

## Author Contributions

JF, LM, JM, and AB designed the work and collected and analyzed the data. All authors contributed to the writing of the manuscript.

## Funding

We acknowledge the funding from “Coordenação de Aperfeiçoamento de Pessoal de Nível Superior” (CAPES; Programa Ciências do Mar, Brazil; grant #84/2010) and “Conselho Nacional de Desenvolvimento Científico e Tecnológico” (CNPq; Instituto Nacional de Ciência e Tecnologia de Toxicologia Aquática, Brazil; grant #573949/2008-5). Adalto Bianchini is research fellow of CNPq (Proc. #307647/2016-1) supported by “International Development Research Center” (IDRC; Ottawa, Canada). Juliana da Silva Fonseca was a graduate fellow from CAPES.

## Conflict of Interest

The authors declare that the research was conducted in the absence of any commercial or financial relationships that could be construed as a potential conflict of interest.

## Publisher’s Note

All claims expressed in this article are solely those of the authors and do not necessarily represent those of their affiliated organizations, or those of the publisher, the editors and the reviewers. Any product that may be evaluated in this article, or claim that may be made by its manufacturer, is not guaranteed or endorsed by the publisher.

## References

[ref1] Banc-Prandi G. Fine M. (2019). Copper enrichment reduces thermal tolerance of the highly resistant Red Sea coral *Stylophora pistillata*. Coral Reefs 38, 285–296. doi: 10.1007/s00338-019-01774-z

[ref2] Beutler E. (1975). “The preparation of red cells for assay,” in Red Cell Metabolism: A Manual of Biochemical Methods (New York, USA: Grune and Straton), 8–18.

[ref3] Bielmyer G. K. Grosell M. Bhagooli R. Baker A. C. Langdon C. Gillette P.. (2010). Differential effects of copper on three species of scleractinian corals and their algal symbionts (*Symbiodinium spp*.). Aquat. Toxicol. 97, 125–133. doi: 10.1016/j.aquatox.2009.12.021, PMID: 20089320

[ref4] Brown G. C. Borutaite V. (2002). Nitric oxide inhibition of mitochondrial respiration and its role in cell death. Free Radic. Biol. Med. 33, 1440–1450. doi: 10.1016/s0891-5849(02)01112-7 12446201

[ref5] Chen Y. Shertzer H. G. Schneider S. N. Nebert D. W. Dalton T. P. (2005). Glutamate cysteine ligase catalysis: dependence on ATP and modifier subunit for regulation of tissue glutathione levels. J. Biol. Chem. 280, 33766–33774. doi: 10.1074/jbc.M504604200, PMID: 16081425

[ref6] CONAMA (2005). Conselho Nacional do Meio Ambiente. Resolução N° 357, de 17 de março de 2005. Brasília, Brazil. Available at: http://www.mma.gov.br/port/conama/res/res05/res35705.pdf (Accessed April, 2021).

[ref7] De Nadal E. Ammerer G. Posas F. (2011). Controlling gene expression in response to stress. Nat. Rev. Genet. 12, 833–845. doi: 10.1038/nrg3055 22048664

[ref9] Dias M. Madeira C. Jogee N. Ferreira A. Gouveia R. Cabral H.. (2019). Oxidative stress on scleractinian coral fragments following exposure to high temperature and low salinity. Ecol. Indic. 107:105586. doi: 10.1016/j.ecolind.2019.105586 31301459

[ref10] Downs C. A. Fouth J. E. Halas J. C. Dustan P. Bemiss J. Woodley C. M. (2002). Oxidative stress and seasonal coral bleaching. Free Radic. Biol. Med. 33, 533–543. doi: 10.1016/s0891-5849(02)00907-3, PMID: 12160935

[ref11] Downs C. A. Fouth J. E. Robinson C. E. Curry R. Lanzendorf B. Halas J.. (2005). Cellular diagnostics and coral health: declining coral health in the Florida keys. Mar. Pollut. Bull. 51, 558–569. doi: 10.1016/j.marpolbul.2005.04.017, PMID: 15992830

[ref12] Downs C. A. Mueller E. Phillips S. Fauth J. E. Woodley C. M. (2000). A molecular biomarker system for assessing the health of coral (*Montastraea faveolata*) During heat stress. Mar. Biotechnol. 2, 533–544. doi: 10.1007/s101260000038, PMID: 14961177

[ref13] Duarte G. Calderon E. N. Pereira C. M. Marangoni L. F. B. Santos H. F. Peixoto R. S.. (2015). A novel marine mesocosm facility to study global warming, water quality, and ocean acidification. Ecol. Evol. 5, 4555–4566. doi: 10.1002/ece3.1670, PMID: 26668722 PMC4670062

[ref14] Dykens J. A. (1984). Enzymic defenses against oxygen toxicity in marine cnidarians containing endosymbiotic algae. Mar. Biol. Lett. 5, 291–301.

[ref15] EPA (2005). United States Environmental Protection Agency. Handbook for Developing Watershed Plans to Restore and Protect Our Waters. EPA 841-B-05-005: Washington. Available at: http://www.epa.gov/owow/nps/watershed_handbook/pdf/handbook.pdf (Accessed April 2021).

[ref16] Fonseca J. S. Marangoni L. F. Marques J. A. Bianchini A. (2017). Effects of increasing temperature alone and combined with copper exposure on biochemical and physiological parameters in the zooxanthellate scleractinian coral *Mussismilia harttii*. Aquat. Toxicol. 190, 121–132. doi: 10.1016/j.aquatox.2017.07.002, PMID: 28709126

[ref17] Fonseca J. S. Marangoni L. F. B. Marques J. A. Bianchini A. (2019). Energy metabolism enzymes inhibition by the combined effects of increasing temperature and copper exposure in the coral M*ussismilia harttii*. Chemosphere 236:124420. doi: 10.1016/j.chemosphere.2019.124420, PMID: 31545208

[ref18] Fonseca J. S. Mies M. Paranhos A. Taniguchi S. Güth A. Z. Bícego M. C.. (2021). Isolated and combined effects of thermal stress and copper exposure on the trophic behavior and oxidative status of the reef-building coral *Mussismilia harttii*. Environ. Pollut. 268:115892. doi: 10.1016/j.envpol.2020.115892, PMID: 33120157

[ref19] Francini-Filho R. B. Cordeiro M. C. Omachi C. Y. Rocha A. M. Bahiense L. Garcia G. D.. (2019). Remote sensing, isotopic composition and metagenomics analyses revealed Doce River ore plume reached the southern Abrolhos Bank reefs. Sci. Total Environ. 697:134038. doi: 10.1016/j.scitotenv.2019.134038, PMID: 32380596

[ref20] Geracitano L. A. Monserrat J. M. Bianchini A. (2002). Physiological and antioxidant enzyme responses to acute and chronic exposure of *Laeonereis acuta* (Polychaeta: nereididae) to copper. J. Exp. Mar. Biol. Ecol. 277, 145–156. doi: 10.1016/S0022-0981(02)00306-4

[ref21] Gissi F. Reichelt-Brushett A. J. Chariton A. A. Stauber J. L. Greenfield P. Humphrey C.. (2019). The effect of dissolved nickel and copper on the adult coral *Acropora muricata* and its microbiome. Environ. Pollut. 250, 792–806. doi: 10.1016/j.envpol.2019.04.030, PMID: 31042619

[ref22] Glynn P. W. (1993). Coral reef bleaching: ecological perspectives. Coral Reefs 12, 1–17. doi: 10.1007/bf00303779

[ref23] Glynn P. W. (1996). Coral reef bleaching: facts, hypotheses and implications. Glob. Chang. Biol. 2, 495–509. doi: 10.1111/j.1365-2486.1996.tb00063.x

[ref24] Grant A. J. Graham K. Frankland S. Hinde R. (2003). Effect of copper on algal-host interactions in the symbiotic coral *Plesiastrea versipora*. Plant Physiol. Biochem. 41, 383–390. doi: 10.1016/S0981-9428(03)00034-2

[ref25] Habig W. H. Jakoby W. B. (1981). Assays for differentiation of glutathione-S-transferase. Methods Enzymol. 77, 398–405. doi: 10.1016/s0076-6879(81)77053-8 7329316

[ref26] Halliwell B. Gutteridge J. (2007). Free Radicals in Biology and Medicine. 4th Edn. New York: Oxford University Press.

[ref27] Hawkins T. D. Bradley B. J. Davy S. K. (2013). Nitric oxide mediates coral bleaching through an apoptotic-like cell death pathway: evidence from a model sea anemone-dinoflagellate symbiosis. FASEB J. 27, 4790–4798. doi: 10.1096/fj.13-235051, PMID: 23934282

[ref28] Hawkins T. D. Davy S. K. (2013). Nitric oxide and coral bleaching: is peroxynitrite generation required for symbiosis collapse? J. Exp. Biol. 216, 3185–3188. doi: 10.1242/jeb.087510, PMID: 23685970

[ref29] Hermes-Lima M. (2004). “Oxygen in biology and biochemistry: role of free radicals,” in Functional Metabolism: Regulation and Adaptation. 1st Edn. ed. Storey K. B.. (New York: John Wiley & Sons), 319–368.

[ref30] Higgins L. G. Hayes J. D. (2011). Mechanisms of induction of cytosolic and microsomal glutathione transferase (GST) genes by xenobiotics and pro-inflammatory agents. Drug Metab. Rev. 43, 92–137. doi: 10.3109/03602532.2011.567391, PMID: 21495793

[ref31] Higuchi T. Fujimura H. Arakaki T. Oomori T. (2009). “Activities of antioxidant enzymes (SOD and CAT) in the coral Galaxea fascicularis against increased hydrogen peroxide concentrations in seawater.” In Proceedings of the 11th International Coral Reef Symposium. July 7-11, 2008. Lauderdale, Florida, 926–930.

[ref32] Higuchi T. Suzuki Y. Fujimura H. (2012). “Multiple effects of hydrogen peroxide and temperature on antioxidants and bleaching.” In Proc 12th Intl Coral Reef Symp. 9–13.

[ref33] Higuchi T. Yuyama I. Nakamura T. (2015). The combined effects of nitrate with high temperature and high light intensity on coral bleaching and antioxidant enzyme activities. Reg. Stud. Mar. Sci. 2, 27–31. doi: 10.1016/j.rsma.2015.08.012

[ref34] Hoegh-Guldberg O. (1999). Climate change, coral bleaching and the future of the world’s coral reefs. Mar. Freshw. Res. 50, 839–866. doi: 10.1071/mf99078

[ref35] Howells E. J. Abrego D. Meyer E. Kirk N. L. Burt J. A. (2016). Host adaptation and unexpected symbiont partners enable reef-building corals to tolerate extreme temperatures. Glob. Chang. Biol. 22, 2702–2714. doi: 10.1111/gcb.13250, PMID: 26864257

[ref36] Hultberg B. Andersson A. Isaksson A. (2001). Interaction of metals and thiols in cell damage and glutathione distribution: potentiation of mercury toxicity by dithiothreitol. Toxicology 156, 93–100. doi: 10.1016/s0300-483x(00)00331-0, PMID: 11164611

[ref37] IPCC (2014). The Fifth Assessment Report of the Intergovernmental Panel on Climate Change (IPCC). Cambridge, UK: Cambridge University Press.

[ref38] Jones R. J. (1997). Zooxanthellae loss as a bioassay for assessing stress in corals. Mar. Ecol. Prog. Ser. 149, 163–171. doi: 10.3354/meps149163

[ref39] Jones R. J. (2004). Testing the ‘photoinhibition’ model of coral bleaching using chemical inhibitors. Mar. Ecol. Prog. Ser. 284, 133–145. doi: 10.3354/meps284133

[ref40] Klein R. D. Borges V. D. Rosa C. E. Colares E. P. Robaldo R. B. Martinez P. E.. (2017a). Effects of increasing temperature on antioxidant defense system and oxidative stress parameters in the Antarctic fish *Notothenia coriiceps* and *Notothenia rossii*. J. Therm. Biol. 68, 110–118. doi: 10.1016/j.jtherbio.2017.02.016, PMID: 28689712

[ref41] Klein R. D. Rosa C. E. Colares E. P. Robaldo R. B. Martinez P. E. Bianchini A. (2017b). Antioxidant defense system and oxidative status in Antarctic fishes: the sluggish rockcod *Notothenia coriiceps* versus the active marbled notothen *Notothenia rossii*. J. Therm. Biol. 68, 119–127. doi: 10.1016/j.jtherbio.2017.02.013, PMID: 28689713

[ref42] Krueger T. Hawkins T. D. Becker S. Pontasch S. Dove S. Hoegh-Guldberg O.. (2015). Differential coral bleaching—contrasting the activity and response of enzymatic antioxidants in symbiotic partners under thermal stress. Comp. Biochem. Phys. 190, 15–25. doi: 10.1016/j.cbpa.2015.08.012, PMID: 26310104

[ref43] Kuhl M. Cohen Y. Dalsgaard T. Jorgensen B. B. Revsbech N. P. (1995). Microenvironment and photosynthesis of zooxanthellae in scleractinian corals studied with microsensors for O2, pH and light. Mar. Ecol. Prog. Ser. 117, 159–172. doi: 10.3354/meps117159

[ref44] Leary S. C. Winge D. R. Cobine P. A. (2009). “Pulling the plug” on cellular copper: the role of mitochondria in copper export. Biochim. Biophys. Acta 1793, 146–153. doi: 10.1016/j.bbamcr.2008.05.002, PMID: 18522804 PMC4021392

[ref45] Lesser M. P. (1997). Oxidative stress causes coral bleaching during exposure to elevated temperatures. Coral Reefs 16, 187–192. doi: 10.1007/s003380050073

[ref46] Lesser M. P. (2006). Oxidative stress in marine environments: biochemistry and physiological ecology. Annu. Rev. Physiol. 68, 253–278. doi: 10.1146/annurev.physiol.68.040104.110001, PMID: 16460273

[ref47] Lesser M. P. Stochaj W. R. Tapley D. W. Shick J. M. (1990). Bleaching in coral reef anthozoans: effects of irradiance, ultraviolet radiation, and temperature on the activities of protective enzymes against active oxygen. Coral Reefs 8, 225–232. doi: 10.1007/bf00265015

[ref48] Maher P. (2005). The effects of stress and aging on glutathione metabolism. Ageing Res. Rev. 4, 288–314. doi: 10.1016/j.arr.2005.02.005 15936251

[ref49] Maher P. (2018). Potentiation of glutathione loss and nerve cell death by the transition metals iron and copper: implications for age-related neurodegenerative diseases. Free Radic. Biol. Med. 115, 92–104. doi: 10.1016/j.freeradbiomed.2017.11.015, PMID: 29170091

[ref50] Marangoni L. F. B. Dalmolin C. Marques J. A. Klein R. D. Abrantes D. Pereira C. M.. (2019a). Oxidative stress biomarkers as potential tools in reef degradation monitoring: a study case in a South Atlantic reef under influence of the 2015-2016 El Niño/southern oscillation (ENSO). Ecol. Indic. 106:105533. doi: 10.1016/j.ecolind.2019.105533

[ref51] Marangoni L. F. B. Marques J. A. Duarte G. A. S. Pereira C. M. Calderon E. N. Castro C. B.. (2017). Copper effects on biomarkers associated with photosynthesis, oxidative status and calcification in the Brazilian coral *Mussismilia harttii* (Scleractinia, Mussidae). Mar. Environ. Res. 130, 248–257. doi: 10.1016/j.marenvres.2017.08.002, PMID: 28823595

[ref52] Marangoni L. F. B. Mies M. Guth A. Z. Banha T. N. S. Inague A. Fonseca J. S.. (2019b). Peroxynitrite generation and increased heterotrophic capacity are linked to the disruption of the coral-Dinoflagellate symbiosis in a scleractinian and hydrocoral species. Microorganisms 7:426. doi: 10.3390/microorganisms7100426, PMID: 31600926 PMC6843776

[ref53] Maria V. L. Bebianno M. J. (2011). Antioxidant and lipid peroxidation responses in *Mytilusgallo provincialis* exposed to mixtures of benzo(a)pyrene and copper. Comp. Biochem. Phys. 154, 56–63. doi: 10.1016/j.cbpc.2011.02.004 21354328

[ref54] Marques J. A. Costa P. G. Marangoni L. F. Pereira C. M. Abrantes D. P. Calderon E. N.. (2019). Environmental health in southwestern Atlantic coral reefs: geochemical, water quality and ecological indicators. Sci. Total Environ. 651, 261–270. doi: 10.1016/j.scitotenv.2018.09.154, PMID: 30236843

[ref55] McCord J. M. Fridovich I. (1969). Superoxide dismutase: an enzymatic function for erythocuprein (hemocuprein). J. Biol. Chem. 244, 6049–6055. doi: 10.1016/S0021-9258(18)63504-5 5389100

[ref56] McWilliams J. P. Cote I. M. Gill J. A. Sutherland W. J. Watkinson A. R. (2005). Acceler-ating impacts of temperature-induced coral bleaching in the Caribbean. Ecology 86, 2055–2060. doi: 10.1890/04-1657

[ref57] Mercer J. F. Llanos R. M. (2003). Molecular and cellular aspects of copper transport in developing mammals. J. Nutr. 133, 1481S–1484S. doi: 10.1093/jn/133.5.1481S, PMID: 12730448

[ref58] Mies M. Francini-filho R. B. Zilberberg C. Garrido A. G. Longo G. O. Laurentino E.. (2020). South Atlantic coral reefs are major global warming refugia and less susceptible to bleaching. Front. Mar. Sci. 7:514. doi: 10.3389/fmars.2020.00514

[ref59] Mitchelmore C. L. Verde E. A. Weis V. M. (2007). Uptake and partitioning of copper and cadmium in the coral *Pocillopora damicornis*. Aquat. Toxicol. 85, 48–56. doi: 10.1016/j.aquatox.2007.07.015, PMID: 17804091

[ref60] Monteiro D. A. Almeida J. A. Rantin F. T. Kalinin A. L. (2006). Oxidative stress biomarkers in the freshwater characid fish, *Brycon cephalus*, exposed to organophosphorus insecticide Folisuper 600 (methyl parathion). Comp. Biochem. Phys. 143, 141–149. doi: 10.1016/j.cbpc.2006.01.004, PMID: 16546452

[ref61] Nadella S. D. Fitzpatrick J. L. Franklin N. Bucking C. Smith S. Wood C. M. (2009). Toxicity of dissolved cu, Zn, Ni and cd to developing embryos of the blue mussel (*Mytilus trossolus*) and the protective effect of dissolved organic carbon. Comp. Biochem. Phys. 149, 340–348. doi: 10.1016/j.cbpc.2008.09.001, PMID: 18832046

[ref62] Nagalakshmi N. Prasad M. N. V. (2001). Responses of glutathione cycle enzymes and glutathione metabolism to copper stress in *Scenedesmus bijugatus*. Plant Sci. 160, 291–299. doi: 10.1016/s0168-9452(00)00392-7, PMID: 11164601

[ref63] Negri A. P. Hoogenboom M. O. (2011). Water contamination reduces the tolerance of coral larvae to thermal stress. PLoS One 6:e19703. doi: 10.1371/journal.pone.0019703, PMID: 21589934 PMC3092768

[ref64] Perez S. Weis V. (2006). Nitric oxide and cnidarian bleaching: an eviction notice mediates breakdown of a symbiosis. J. Exp. Biol. 209, 2804–2810. doi: 10.1242/jeb.02309, PMID: 16809471

[ref65] Portner H. O. (2001). Climate change and temperature dependent biogeography: oxygen limitation of thermal tolerance in animals. Naturwissenschaften 88, 137–146. doi: 10.1007/s001140100216, PMID: 11480701

[ref66] Portner H. O. (2002). Climate variations and the physiological basis of temperature dependent biogeography: systemic to molecular hierarchy of thermal tolerance in animals. Comp. Biochem. Phys. 132, 739–761. doi: 10.1007/s001140100216, PMID: 12095860

[ref67] Pourahmad J. O’Brien P. J. (2000). A comparison of hepatocyte cytotoxic mechanisms for Cu^2+^ and Cd^2+^. Toxicology 143, 263–273. doi: 10.1016/s0300-483x(99)00178-x, PMID: 10755712

[ref68] Rainbow P. S. (2002). Trace metal concentrations in aquatic invertebrates: why and so what? Environ. Pollut. 120, 497–507. doi: 10.1016/s0269-7491(02)00238-5, PMID: 12442773

[ref69] Ralph P. J. Gademann R. Larkum A. W. (2001). Zooxanthellae expelled from bleached corals at 33 °C are photosynthetically competent. Mar. Ecol. Prog. Ser. 220, 163–168. doi: 10.3354/MEPS220163

[ref70] Regoli F. Giuliani M. E. (2014). Oxidative pathways of chemical toxicity and oxidative stress biomarkers in marine organisms. Mar. Environ. Res. 93, 106–117. doi: 10.1016/j.marenvres.2013.07.006 23942183

[ref71] Schwarz J. A. Mitchelmore C. L. Jones R. O’dea A. Seymour S. (2013). Exposure to copper induces oxidative and stress responses and DNA damage in the coral *Montastraea franksi*. Comp. Biochem. Phys. 157, 272–279. doi: 10.1016/j.cbpc.2012.12.003, PMID: 23268349

[ref72] Smith D. J. Suggett D. J. Baker N. R. (2005). Is photoinhibition of zooxanthellae photosynthesis the primary cause of thermal bleaching in corals? Glob. Change Biol. 11, 1–11. doi: 10.1111/j.1529-8817.2003.00895.x

[ref73] Suggett D. J. Smith D. J. (2019). Coral bleaching patterns are the outcome of complex biological and environmental networking. Glob. Change Biol. 26, 68–79. doi: 10.1111/gcb.14871, PMID: 31618499

[ref74] Townsend D. M. Tew K. D. Tapiero H. (2003). The importance of glutathione in human disease. Biomed. Pharmacother. 57, 145–155. doi: 10.1016/s0753-3322(03)00043-x 12818476 PMC6522248

[ref75] van Dam J. W. Negri A. P. Uthicke S. Mueller J. F. (2011). “Chemical pollution on coral reefs: exposure and ecological effects,” in Ecological Impacts of Toxic Chemicals. eds. Sánchez-Bayo F. Brink P. J. Mann R. M. (Amsterdam: Bentham Science Publishers).

[ref76] Weis V. M. (2008). Cellular mechanisms of cnidarian bleaching: stress causes the collapse of symbiosis. J. Exp. Biol. 211, 3059–3066. doi: 10.1242/jeb.009597, PMID: 18805804

[ref77] White C. C. Viernes H. Krejsa C. M. Botta D. Kavanagh T. J. (2003). Fluorescence based microtiter plate assay for glutamate-cysteine ligase activity. Anal. Biochem. 318, 175–180. doi: 10.1016/s0003-2697(03)00143-x, PMID: 12814619

[ref78] Wink D. A. Mitchell J. B. (1998). Chemical biology of nitric oxide: insights into regulatory, cytotoxic, and cytoprotective mechanisms of nitric oxide. Free Radic. Biol. Med. 25, 434–456. doi: 10.1016/s0891-5849(98)00092-6, PMID: 9741580

[ref79] Yakovleva I. M. Baird A. H. Yamamoto H. H. Bhagooli R. Nonaka M. Hidaka M. (2009). Algal symbionts increase oxidative damage and death in coral larvae at high temperatures. Mar. Ecol. Prog. Ser. 378, 105–112. doi: 10.3354/meps07857

[ref80] Yuyama I. Ito Y. Watanabe T. Hidaka M. Suzuki Y. Nishida M. (2012). Differential gene expression in juvenile polyps of the coral *Acropora tenuis* exposed to thermal and chemical stresses. J. Exp. Mar. Biol. Ecol. 430-431, 17–24. doi: 10.1016/j.jembe.2012.06.020

