# Supplementary material for: Elevated Temperature and Exposure to Copper Leads to Changes in the Antioxidant Defense System of the Reef-Building Coral Mussismilia harttii
Source: Front Physiol. 2021 Dec 23;12:804678. doi: 10.3389/fphys.2021.804678 (PMC8734030; doi:10.3389/fphys.2021.804678)
Supplement: Supplementary file 1 [file Table_1.docx]

Table S1: Results physicochemical parameters of experimental seawater during the exposure of the coral *Mussismilia harttii* to three different temperatures and three different copper (Cu) concentrations for 4 and 12 days. All the physiochemical parameters (salinity, pH, pluviometry, temperature) represent an average of four and twelve days of data, except for DOC, in which water samples were collected every 3 days.

| Physicochemical parameters | 4 days | 12 days |
| --- | --- | --- |
| Dissolved organic carbon (mg L⁻¹) | 3.35 ± 0.35 | 1.36 ± 0.46 |
| Salinity (ppt) | 35.5 ± 0.06 | 36.9 ± 0.08 |
| pH | 8.29 ± 0.00 | 8.29 ± 0.00 |
| Pluviometry (mm) | 0.23 ± 0.05 | 0.01 ± 0.00 |
| Temperature (°C) | 25.2 ± 0.05 | 25.5 ± 0.03 |
